# Partitioning of diet between species and life history stages of sympatric and cryptic snappers (Lutjanidae) based on DNA metabarcoding

**DOI:** 10.1038/s41598-020-60779-9

**Published:** 2020-03-09

**Authors:** Miwa Takahashi, Joseph D. DiBattista, Simon Jarman, Stephen J. Newman, Corey B. Wakefield, Euan S. Harvey, Michael Bunce

**Affiliations:** 1 School of Molecular and Life Sciences, Curtin University, Kent Street, Bentley, Perth, WA 6102 Australia; 2 Australian Museum Research Institute, Australian Museum, 1 William Street, Sydney, NSW 2010 Australia; 3 School of Biological Sciences, University of Western Australia, 35 Stirling Highway, Perth, WA 6009 Australia; 4 Western Australian Fisheries and Marine Research Laboratories, Department of Primary Industries and Regional Development, Government of Western Australia, P.O. Box 20, North Beach, WA 6920 Australia; 5 Environmental Protection Authority, 215 Lambton Quay, Wellington, 6011 New Zealand

**Keywords:** Community ecology, Ecological networks, Molecular ecology, Ecology

## Abstract

*Lutjanus erythropterus* and *L. malabaricus* are sympatric, sister taxa that are important to fisheries throughout the Indo-Pacific. Their juveniles are morphologically indistinguishable (i.e. cryptic). A DNA metabarcoding dietary study was undertaken to assess the diet composition and partitioning between the juvenile and adult life history stages of these two lutjanids. Major prey taxa were comprised of teleosts and crustaceans for all groups except adult *L. erythropterus*, which instead consumed soft bodied invertebrates (e.g. tunicates, comb jellies and medusae) as well as teleosts, with crustaceans being notably absent. Diet composition was significantly different among life history stages and species, which may be associated with niche habitat partitioning or differences in mouth morphology within adult life stages. This study provides the first evidence of diet partitioning between cryptic juveniles of overlapping lutjanid species, thus providing new insights into the ecological interactions, habitat associations, and the specialised adaptations required for the coexistence of closely related species. This study has improved our understanding of the differential contributions of the juvenile and adult diets of these sympatric species within food webs. The diet partitioning reported in this study was only revealed by the taxonomic resolution provided by the DNA metabarcoding approach and highlights the potential utility of this method to refine the dietary components of reef fishes more generally.

## Introduction

Reef fish communities are extraordinarily diverse^1^, and there is often partitioning of resources, particularly for food and habitat, between sympatric reef fishes^2–4^. This ecological process, called niche partitioning, is fundamental for the coexistence of species within an ecosystem^5^. Niche partitioning is especially relevant among species of the same genus, which may include cryptic and sympatric species, because the more similar the co-existing species are, the more intensively they presumably compete^2,4,6^. Niche partitioning within the same species is also a common strategy in reef fish in order to minimise intra-specific competition, and can be associated with ontogenetic movements relative to life history stages. Indeed, the life cycle of many reef fishes consists of a pelagic larval phase, followed by a demersal juvenile phase on shallow, low-relief substrate (e.g. mangrove and seagrass nurseries), and an adult phase on deeper, high-relief reefs^7,8^. Ontogenetic dietary shifts were previously identified in a number of snapper species (family Lutjanidae)^9–11^, suggesting that habitat partitioning between juvenile and adult fish was based not only on finding refuge from predation, but also for accessing food resources^12,13^.

*Lutjanus erythropterus* (Bloch, 1790) and *L. malabaricus* (Bloch & Scheneider, 1801) are sympatric snapper species that co-exist in the tropical and subtropical Indo-Pacific region^14^. They are sister taxa^15^, and the juveniles are phenotypically cryptic^8^. Both species support important commercial and recreational fisheries on tropical and subtropical coasts throughout their geographic distribution^14,16^. Diet compositions of fishery targeted species at all life history stages and their ecological interactions are important considerations for ecosystem based fisheries management^17,18^. Due to their similarities in morphology, ecology, and the nature of their fisheries, they are often categorised as “red snappers” and are combined into a single species group within catch data in some parts of the world^16,19^. Moreover, despite their importance to fisheries, little is known about their ecological interactions and niche partitioning^16,20,21^. Common dietary items visually identified from stomach contents in previous studies include crustaceans, teleosts and cephalopods^20,22^. Despite the length range of *L. erythropterus* and *L. malabaricus* examined in these dietary studies being from 38 to 570 mm, the diet partitioning between the species and life history stages has never been examined.

Dietary partitioning between morphologically similar species, such as *L. erythropterus* and *L. malabaricus*, requires precise dietary analysis methods because these two predators are likely to be ecologically similar and exhibit subtle, if any, differentiation^3,4,6^. Previous studies identified high levels of dietary overlap between some of the coral-feeding, sympatric butterflyfishes (family Chaetodontidae) using an *in-situ* feeding observation method^23,24^. Niche overlap and coexistence are possible when the population sizes are not limited by the availability of shared resources. However, Nagelkerken *et al*.^4^ identified clear dietary partitioning between 21 species of butterflyfish by visually examining their gut contents, whereas other methods (*in-situ* feeding observations and stable isotope analyses) did not detect such partitioning. These conflicting results suggest that some dietary analysis methods may lack the resolution needed to detect distinct, and sometimes subtle, differences in diet^4,25^. Species-level identification of prey items is now possible through the use of DNA metabarcoding^26–28^. DNA metabarcoding simultaneously generates millions of copies of DNA sequences of digested prey from predators’ gut contents or faeces, and matches them against barcode sequences in databases to reveal the taxa of consumed species^6,29^. This method lends itself to more comprehensive evaluation of dietary partitioning of sympatric, cryptic species at a much finer-scale^30,31^.

In this study, we conducted metabarcoding-based dietary analyses to assess the diet composition of juvenile and adult *L. erythropterus* and *L. malabaricus*. We hypothesised that there would be significant dietary partitioning between species to reduce inter-specific competition. Dietary partitioning relating to developmental stage is also a reasonable explanation for coexistence by reducing intra-specific competition through ontogenetic habitat shifts, which have been previously observed in other lutjanid species^9–13^. With the aid of DNA metabarcoding, we expected to identify a variety of prey taxa at lower taxonomic levels to test these hypotheses. Information on how diet varies between sister taxa and life history stages will improve our understanding of the ecological processes allowing them to coexist in marine ecosystems.

## Materials and Methods

### Sample collection

**Figure 1:**
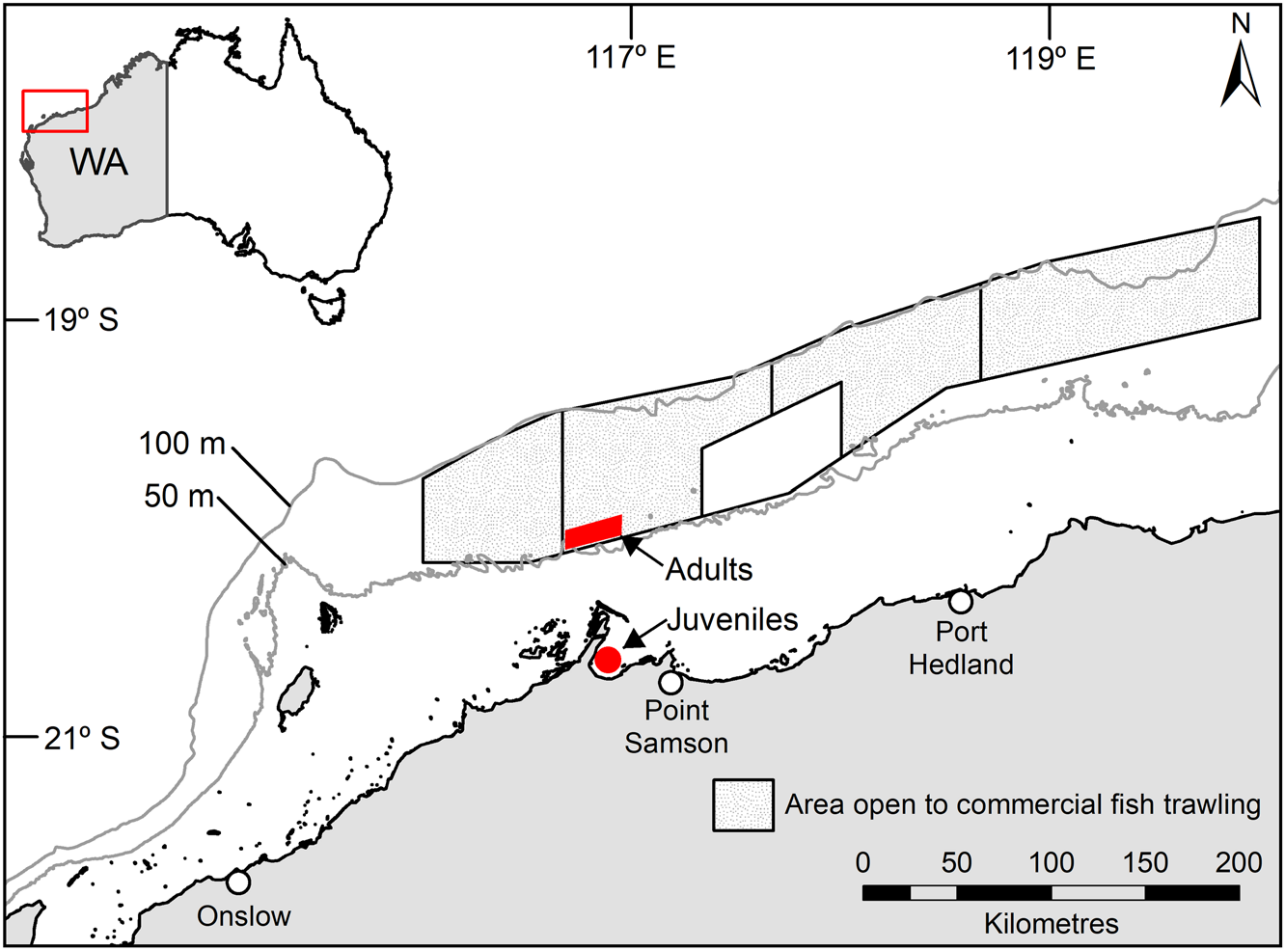
Locations where juvenile and adult samples of *Lutjanus erythropterus* and *L. malabaricus* were collected in the Pilbara region of north-western Australia (generated using ArcMap v10.3.1, https://desktop.arcgis.com). Areas open to commercial fish trawling (shaded) and the 50 m and 100 m depth contours (grey lines) are shown.

Samples of juvenile and adult *L. erythropterus* and *L. malabaricus* were caught using demersal trawls from the Pilbara region of north-western Australia (Fig. 1). The use of this fishing method mitigated the potential for fish to consume bait during capture, which may confound natural diet compositions. In addition, to reduce potential biases that may be associated with temporal and spatial variations in diet composition, juveniles and adults were sampled from the same trawl catches. However, considering juveniles and adults occupy different habitats for both species, the two life stages were sampled within as close proximity as practical. Juvenile *L. erythropterus* and *L. malabaricus* (99 to 201 mm total length, TL) were sampled during a research survey from depths of 9–24 m within a nearshore marine embayment (i.e. Nicol Bay) in July and August 2017 (Table 1 & Fig. 1, see trawl net configuration in Wakefield *et al*.^32^). Concurrently, catches of adult *L. erythropterus* and *L. malabaricus* (482 to 795 mm TL) taken by commercial fishers using demersal fish trawls (see trawl net configuration in Wakefield *et al*.^33^) in 52–58 m depth and ~60 km directly offshore from where the juveniles were sampled (Table 1 & Fig. 1), were labelled and kept separate from the rest of the catch before collection approximately three days later by research staff. All fish samples were frozen whole (−20 °C) prior to dissection under laboratory conditions to avoid potential contamination. The TL of each fish was measured to the nearest 1 mm.

**Table 1:** Number of juvenile and adult *Lutjanus erythropterus* (LE) and *L. malabaricus* (LM) for each category of sampling variables. Fullness of stomach was recorded as “full” when a prey item was observed in a stomach.

Variables	Levels	Juvenile		Adult	
		LE	LM	LE	LM
Fullness of stomach	Empty	8	4	10	7
	Full	3	7	1	5
TL (mm)	50–100	1	0		
	100–150	9	8		
	150–200	1	2		
	200–250	0	1		
	450–500			3	
	500–550			8	
	550–600				1
	650–700				6
	700–750				1
	750–800				4
Date	14-Jul-17	6	6		
	13-Aug-17		1		
	18-Aug-17	5	4	11	12
Time	00:01–03:00		1		
	06:01–09:00	1			
	09:01–12:00	1	2		
	15:01–18:00	3	3		
	18:01–21:00	6	2		
	21:01–24:00		3		
	03:30–22:40			11	12
Depth (m)	8.1 to 9	1			
	9.1 to 10		1		
	10.1 to 11		2		
	11.1 to 12	7	4		
	12.1 to 13	3	3		
	23.1 to 24		1		
	52–58			11	12

Sampling details such as time and depth were not available for each adult specimen as trawl shot numbers were not recorded for individual fish.

### Species identification of cryptic juveniles

Considering juveniles of *L. erythropterus* and *L. malabaricus* are cryptic, fin clips were collected and stored in 99% ethanol in order to genetically identify the species via DNA barcoding. DNA of the tissues was extracted, diluted 1/10, and amplified following the “HotSHOT” technique described by Meeker *et al*.^34^. Polymerase chain reaction (PCR) was performed using FishBCH forward primer (5′-ACTTCYGGGTGRCCRAARAATCA-3′) and FishBCL reverse primer (5′-TCAACYAATCAYAAAGATATYGGCAC-3′) to target 600 to 800 bp of the cytochrome c oxidase subunit I (COI) region of the mitochondrial DNA. The PCR cycling program used was: (i) 94 °C for 4 minutes, (ii) 35 amplification cycles of 94 °C for 30 seconds, 50 °C for 30 seconds, and 72 °C for 60 seconds, and (iii) a final extension step at 72 °C for 10 minutes. Following amplification, 4 µL of amplicons were loaded onto a 2% agarose gel, and the gel image was analysed under UV light using a Bio-Rad transilluminator and GelRed nucleic acid staining dye (Molecular Probes) in order to ensure the successful amplification at the targeted size. DNA with no signature on the gel image was further diluted 1/25, re-amplified, and visualised on a gel. Two µL of exonuclease I and FastAP thermosensitive alkaline phosphatase were added to 10 µL of the PCR product and purified using the following thermocycler cycling program; (i) 37 °C for 15 minutes, (ii) 80 °C for 15 minutes, and (iii) 4 °C for 10 minutes. The purified amplicons were shipped to Macrogen for Sanger Sequencing (Macrogen Facility, Seoul, Korea) in the forward direction. Sequencing data were queried against the National Center for Biotechnology Information (NCBI) GenBank nucleotide reference database^35^ and an in-house fish database^36^ using the Basic Local Alignment Search Tool (BLASTn)^37^. All sequences were assigned to either *L. erythropterus* or *L. malabaricus* based on percentage similarities scores.

### Gastrointestinal tract content dissection

Juvenile and adult *L. erythropterus* and *L. malabaricus* (n = 11~13 for each species and life history stage) were thawed at room temperature prior to measurement and dissection. New, sterile surgical blades and gloves were used for each fish, and the other dissection utensils were cleaned using bleach and ethanol and exposed to UV for a minimum of 20 minutes between each sample to minimise cross-contamination. Entire gastrointestinal tracts (GIT) were removed from host fish. Intestinal content was collected in a separate sample container. Intestinal contents were used instead of stomach contents in this study as non-prey tissues might have been ingested during trawl capture events. Fullness of stomach was recorded in binary format – “full” or “empty” if prey items were present or absent, respectively (Table 1).

### DNA extraction

Each intestinal content sample of adult fish was homogenised using Omni Hard Tissue Tip homogeniser, and between 150 and 250 mg of homogenised content was subsampled into a 5 mL tube. Homogenising and subsampling steps were omitted for juvenile intestinal samples due to the low volume of material obtained from each juvenile fish. DNA from the GIT sample was extracted using QIAamp PowerFecal DNA Kits following the manufacturer’s instructions. This extraction kit was used in this study as it was designed to effectively remove PCR inhibitors often present in gut content and faecal samples. DNA extraction controls consisting of the extraction reagents but no sample were processed concurrently for each set of extractions.

### Amplification and sequencing

DNA extracts of intestinal contents were amplified and sequenced separately for each sample. In order to determine the optimal dilution of DNA extracts specific to each sample and each assay, quantitative PCR (qPCR) was performed on the neat and 1/10 dilution of DNA extracts with a range of universal and taxa-specific assays (Table 2), and amplification efficiency and inhibition was examined by the C_T_ values and amplification curves. qPCR was performed following the PCR mixture formula and cycling program described in Berry *et al*.^38^, except for the PCR mixture containing Fish16S primers. One µL of species-specific blocking primers (LMBP or LEBP, depending on the host species) (100 µM) was added into the PCR mixture containing Fish16S primers in order to suppress the amplification of host DNA (see Supplementary ‘Blocking primer development’, Table S1, Figs. S1–S3).

**Table 2:** List of assays targeting genes of potential prey items of *Lutjanus erythropterus* and *L. malabaricus*, and host-specific blocking primer (LEBP, *L. erythropterus* blocking primer; LMBP, *L. malabaricus* blocking primer).

Assay name	Assay sequence (5′-3′)	Target taxa	Target gene	T (°C)	Product size (bp)	Minimum length (bp)	Reference
18 SUni (F)	GCCAGTAGTCATATGCTTGTCT	Universal	18S	52	350–420	200	^66^
18SUni (R)	GCCTGCTGCCTTCCTT						^66^
SCeph (F)	GCTRGAATGAATGGTTTGAC	Cephalopods	16S	50	90–110	50	^67^
SCeph (R)	TCAWTAGGGTCTTCTCGTCC						^67^
SCrust (F)	GGGACGATAAGACCCTATA	Crustaceans	16S	51	140–190	100	^47^
SCrust (R)	ATTACGCTGTTATCCCTAAAG						^47^
Fish16S (F)	GACCCTATGG***AGCTTTAGAC***	Teleosts	16S	58	200	100	^47^
Fish16S (R)	CGCTGTTATCCCTADRGTAACT						^26^
LMBP	***AGCTTTAGAC***ACCAAGGCAGACCAT/C3/	*L. malabaricus*	16S	58	N/A	N/A	This study
LEBP	***AGCTTTAGAC***ACCAAGGCAGAACAT/C3/	*L. erythropterus*					This study

The first 10 base pairs of the blocking primers overlap with the 3′ end of the forward Fish16S assay (bold and italic font), followed by 15 base pairs of the host specific sequences. The C3 spacer at the 3′ end is a modified DNA oligonucleotide, which inhibits annealing. Minimum length of each assay is the threshold length used for quality filtering of sequences. T, annealing temperature; F, forward primer; R, reverse primer.

For metabarcoding using an Illumina MiSeq platform (V2 chemistry), fusion PCRs were then performed on DNA extracts (with appropriate DNA input determined by qPCR) for each fusion assay with MID tags – these PCRs followed the same reaction conditions and cycling program used in qPCR. To detect and minimise contamination, undiluted extraction controls, as well as the PCR controls containing the PCR mixture with no DNA extracts were also amplified with the MID tags of each assay during the fusion PCR. Each sample was run in duplicate with the same MID tag in order to mitigate PCR stochasticity and ensure a sufficient concentration of amplicons was available for library pooling and quantitation. DNA library preparation and sequencing were performed, following the technique described in Berry *et al*.^38^. All the laboratory procedures were carried out in the Trace and Environmental DNA (TrEnD) Laboratory at Curtin University in Western Australia.

### Quality filtering of sequence reads and taxonomic assignment to prey taxa

The sequencing output files were downloaded in FASTQ format, and assigned to samples by finding sample-specific MID tags and gene-specific assays using the DNABarcodes package in R^39^. Sequencing adapter, MID tags, and gene-specific assays were annotated and trimmed. Quality filtering was run on the trimmed sequences using DADA2 R package^40^ with the following criteria: (1) removal of reads shorter than assay-specific minimum length thresholds (Table 2), and (2) removal of reads with the number of expected errors more than 1 for single-end reads and the forward reads of paired-end sequences, and 2 for reverse reads of paired-end sequences. Higher maximum number of expected errors (maxEE; less stringent cut-off) was allowed for reverse reads of paired-end sequences because the quality of reverse reads is generally poorer than those of forward reads in Illumina paired-end sequencing technology^41,42^. Indeed, the quality profile plots produced in the DADA2 pipeline demonstrated that our reverse reads had lower quality scores towards the end of the cycles compared to forward reads (see Supplementary Fig. S4). Filtered sequences were dereplicated and singletons and chimeric sequences were removed. Paired end sequences were merged with the minimum overlap of 20 bases. Denoised, exact sequence variants constructed using this protocol are called amplicon sequence variants (ASVs)^43^. ASV tables were created for each assay^43^, and a BLASTn search was carried out against the NCBI GenBank nucleotide reference databases and an in-house fish database^36^ to assign ASVs to taxa^35^, allowing 100% coverage matching and a minimum of 95% identity matching.

The reference sequences with the highest identity matching to query sequences were called primary reference sequences. When the difference between the percent identity matches of primary and non-primary reference sequences was more than a set similarity threshold, the non-primary reference sequences were omitted. In other words, the threshold defined the maximum difference between the percent identity matches of primary and non-primary reference sequences allowed in the lowest common ancestor (LCA) assignment algorithm. The threshold of 0, 1 and 2% were used initially in order to examine how the threshold selection would affect the results of our study. 0% threshold provided the highest proportion of taxonomic assignments, whereas 1 and 2% thresholds provided equivalent taxonomic assignments (see Supplementary Table S2-A). The statistical significance of diet partitioning patterns remained unchanged with different thresholds (see Supplementary Table S2-B, C). Following these comparisons, the 1% threshold was selected primarily because it reduced potential artefact taxa and was no different to the 2% threshold assignments. When there was more than one species assigned to an ASV, the taxonomic level was dropped to lower levels (i.e. genus, family) in order to assign the LCA. For instance, if one unique ASV was assigned to multiple species from the same genus, it was assigned to the genus of those species. If the assigned species were from different genera but the same family, the taxonomic assignment was dropped to the family level. When the taxonomic level of LCA was class or above, the ASV was removed. The LCA assignment was performed using the in-house script developed by M. Mousavi (unpublished).

Terrestrial fauna, flora and fungi (e.g. human, flowering plants) were considered to be environmental contaminants and removed from the list of the assigned taxa. The following marine taxa were also assumed to be non-prey items and removed from the list of prey taxa; parasitic organisms commonly found on fish, algae, dinoflagellates, ciliates, unicellular flagellate eukaryotes, and the host species. Three ASVs were detected from extraction controls using 18SUni assays. Two of them (both assigned to *Penaeus vannamei*) were only detected from one control sample and not from any of the intestine samples, whereas one ASV (assigned to family Pomacanthidae using LCA) was detected in all five extraction controls as well as all intestine samples using the 18SUni assay, accordingly, the ASV was removed from the prey item list.

### Data analyses

The number of samples and sequencing depth were examined to inspect whether our sampling and sequencing efforts were sufficient to capture the majority of their potential prey taxa (see Supplementary ‘Assessment of sampling and sequencing depth’, Figs. S5–S7). The mean number of reads and prey taxa obtained from each life history stage and species were compared using analysis of variance (ANOVA).

Studies using metabarcoding, including dietary studies, can interpret the relative read abundance (RRA) as a proxy for relative abundance of taxa based on positive correlations between RRA and independent measures of abundance^44–46^. On the other hand, presence/absence (PA) transformation of sequence reads is also commonly applied in eDNA studies due to the various sources of RRA bias, such as differences in template DNA abundance, marker choice, and the different states of digestion of prey material^25,44,47^. RRA data generated with universal primers, including the 18SUni primer used in this study, are particularly biased due to the mismatches in the primer binding regions and amplification of diverse multi-template mixtures including non-target species, such as unicellular eukaryotes^48,49^. Furthermore, Piñol *et al*.^48^ suggested that blocking primers could co-block non-target species with as little as four mismatches during PCR reactions, suggesting the limitation of a quantitative analysis. Given these limitations in quantitative approaches, we carried out multivariate analyses using PA data only, while further study using RRA might be possible using other assays and a more controlled experimental design. Jaccard coefficient matrices were constructed from the LCA table with PA datasets^50^. The difference in the diet compositions between the species and life history stages were statistically tested using two-way permutational multivariate analysis of variance (PERMANOVA) with 9999 permutations, followed by pairwise PERMANOVA due to a significant interaction term. Fullness of stomach was incorporated in PERMANOVA as a covariate in order to test whether the diet composition was affected by the timing of recent feeding events and fish sampling. The similarity matrices were visualised using Canonical Analysis of Principal Coordinates (CAP) ordination plot^51^. Allocation success rates on CAP plots were estimated using the “leave-one-out” approach. Trace and delta canonical test statistics were also obtained using 9999 permutations in order to support the results of PERMANOVA. Distance-based linear model (DistLM) analysis was undertaken in order to test the interaction effects between sample variability and diet composition of juvenile fish. The factors tested in DistLM included species and TL, and various sampling factors such as time, date and depth of sampling (Table 1). Detailed sampling information is not available for adult fish samples as trawl shot numbers were not recorded for individual fish, thus DistLM was not carried out for the adult fish samples. BEST routine and Akaike Information Criterion values for finite sample sizes (AICc) were used to select the most parsimonious combination of variables that best explained the diet data^50^. The above multivariate analyses were conducted using the software PRIMER 7 (v. 7.0.13, https://www.primer-e.com/)^50^. All figures were produced using RStudio (v.1.0.143, https://rstudio.com/)^52^.

### Ethics statement

Prior to the commencement of the study ethical approval and guidelines were waived by the Office of Research and Development at Curtin University, WA, Australia, as samples used in this study were collected by staff from the Western Australian Fisheries and Marine Research Laboratories, Department of Primary Industries and Regional Development, Government of Western Australia (DPIRD) for different projects with the appropriate government permits. Frozen samples were provided to the authors after data were collected for different projects.

## Results

A total of 9,389,741 reads were obtained from 48 intestinal samples of juvenile and adult *L. erythropterus* and *L. malabaricus* using four metabarcoding assays selected to profile prey items. A total of 7,166,024 reads (accounting for 76% of the total reads) remained after quality filtering. These reads were dereplicated to a total of 1244 ASVs; 863 ASVs were assigned to taxa, and 179 ASVs were considered as potential primary prey items, which consisted of 37 unique prey taxa.

The number of sequence reads varied between samples, ranging from 3,385 to 301,747 reads. The mean number of reads obtained from each life history stage and species were significantly different (ANOVA: F_3,41_ = 11.6, p <0.0001), juvenile *L. erythropterus* had a significantly higher number of reads than all other groups (see Supplementary Fig. S5-A). The mean number of prey taxa were not significantly different between the groups, ranging from 2.18 (±0.87 SD) to 3.67 (±2.23 SD) (ANOVA: F_3,41_ = 1.31, p = 0.28) (see Supplementary Fig. S5-B). There was no correlation between the number of reads and the prey taxa assigned (Pearson’s correlation: t_43_ = 0.13, p = 0.90). These non-significant results cumulatively indicate that the sequencing effort did not affect the number of prey taxa identified from the intestinal content despite some differences in read depth. This finding was also supported by rarefaction curves, where most curves plateaued between 1000 and 5000 reads, suggesting that sequencing depth was sufficient for our samples to detect the average number of ASV and prey items within an assay (see Supplementary Fig. S6). Therefore, no rarefaction or sequence subsampling was applied to our dataset for further analyses. By contrast, species accumulation curves did not reach a plateau with the number of samples collected for this study, indicating that more prey taxa might have been identified with more sample replicates (see Supplementary Fig. S7).

**Figure 2:**
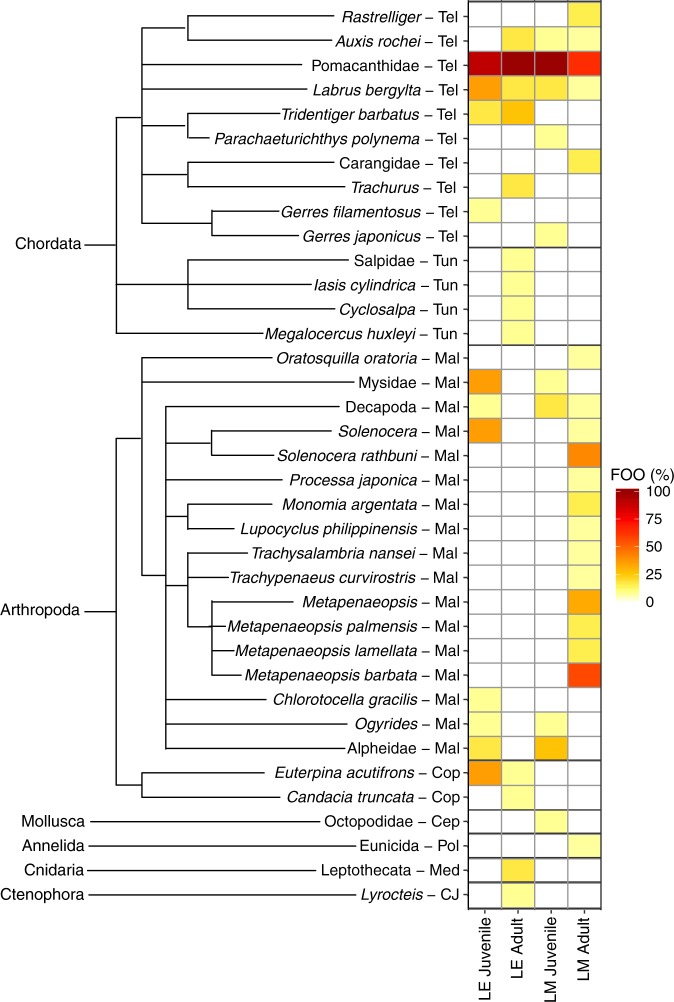
Frequency-of-occurrence (FOO) (%) of prey taxa identified in the intestinal contents of juvenile and adult *Lutjanus erythropterus* (LE) and *L. malabaricus* (LM). Two to three letters at the end of the taxa names refers to general diet categories; Tel, teleost; Tun, tunicate; Mal, malacostracan crustacean; Cop, copepod; Cep, cephalopod; Pol, polychaete; Med, medusa; CJ, comb jelly.

Species level assignment was achieved for 57% of the prey taxa, whereas 43% were dropped from species to higher taxonomic levels using the LCA assignment algorithm; 19%, 16%, and 8% at genus, family, and order levels respectively (see Supplementary Table S3-A). Up to three species from the same genus were assigned to each ASV with the LCA taxa level of genus (see Supplementary Table S3). When LCA taxa levels are family and order, up to 10 and 30 species, respectively, were assigned to the ASV(s) (see Supplementary Table S3). ASVs with the LCA of Decapoda (order) were assigned to 30 species from 14 families, with the percentage similarities ranging between 96.68 and 100% (see Supplementary Table S3). The prey taxa consisted of 6 phyla (Chordata, Arthropoda, Cnidaria, Ctenophora, Annelida and Mollusca), and they were further categorised into eight general diet categories; (i) teleosts, (ii) malacostracan crustaceans, which includes crabs, prawns, mantis shrimps and mysid shrimps, (iii) copepods, (iv) medusa including hydrozoan and scyphozoa that have a free-swimming medusa stage at one point of their life cycle, (v) tunicates, (vi) comb jellies, (vii) polychaetes, and (viii) cephalopods. The majority of the prey taxa were from the groups of teleost and malacostraca (most of which are from the order Decapoda), accounting for 27% and 45% of the total taxa found, respectively (Fig. 2). Two of the 37 assigned taxa (accounting for 5% of the total taxa detected) were shared across all the four groups, all of which were teleosts; Pomacanthidae and *Labrus bergylta* (Fig. 2). Pomacanthidae, the angelfish family, was the most common prey taxa across both species and life stages with high occurrence rates (Fig. 2). Pomacanthidae was the lowest common ancestor (LCA) of 10 species, indicating that multiple species of Pomacanthidae might have been consumed (see Supplementary Table S3). Importantly, 27 out of the 37 assigned taxa (accounting for 73%) were not shared, and occurred in only one of the four groups (Fig. 2).

Diet compositions were significantly different between the species and their life history stages (PERMANOVA: p = 0.0039 and 0.0008 respectively, Table 3A). The effect of stomach fullness on diet composition was not significant (PERMANOVA: p = 0.12, Table 3A). The interaction between the two factors was significant, indicating that the partitioning patterns were not consistent within each factor (PERMANOVA: p = 0.0025, Table 3A). Pairwise PERMANOVA identified the significant inter-specific dietary partitioning within each of the life history stages (p = 0.019 within juvenile, p = 0.027 within adult; Table 3B) and significant intra-specific partitioning within each of the species (p = 0.031 within *L. erythropterus*, p = 0.0019 within *L. malabaricus*; Table 3B).

**Table 3:** The results of PERMANOVA (A) and pairwise-PERMANOVA (B) testing the differences in diet composition of juvenile and adult *Lutjanus erythropterus* (LE) and *L. malabaricus* (LM), using 9999 permutations.

(A) Permanova				
	df	SS	F Model	p
Species	1	6416	2.40	0.0039
Life stage	1	7667	2.87	0.0008
Fullness of stomach	1	3820	1.43	0.12
Sp x Life stage	1	8071	3.02	0.0025
Residuals	40	106770		
**(B) Pairwise-PERMANOVA**				
		**df**	**t**	**p**
Inter-specific	Within Juvenile	19	1.50	0.019
	Within Adult	20	1.43	0.027
Intra-specific	Within LE	19	1.43	0.031
	Within LM	20	1.99	0.0019

Fullness of stomach was incorporated into the analyses as a covariate in order to test its effect on diet composition. The tests were based on Jaccard coefficient matric for presence and absence (PA) datasets.

**Figure 3:**
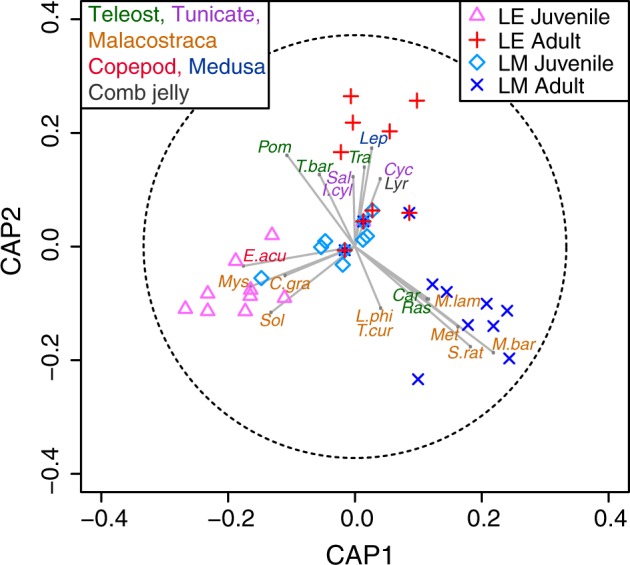
Canonical Analyses of Principal Coordinates (CAP) ordination plots of prey assemblage data from intestinal contents of adult and juvenile *Lutjanus erythropterus* (LE) and *L. malabaricus* (LM), using Jaccard coefficient matrix of presence and absence (PA) data. The overlayed vectors are the Pearson correlations of prey taxa with the two canonical axes, which had the correlation coefficient higher than 0.3. The circle in CAP indicated the correlation coefficient of 1. The closer the vector reached to the circle, the higher the correlation coefficient is. The first three characters of the taxa names were displayed if the taxa were assigned to genus or higher levels. The first character of the genus and three characters of species names are displayed if the taxa were assigned to the species level. Colour of the taxa labels indicate the functional categories.

The two canonical test statistics also identified the significant differences in the diet compositions among the four groups (p = 0.0001 and 0.0026 for trace and delta statistics, respectively, with 9999 permutations), supporting the results of PERMANOVA. The squared canonical correlations of the first and second canonical axes were 0.82 and 0.73. The CAP analysis included 12 PCO axes (m = 12), which achieved the maximum proportion of correct allocations (64%). Leave-one-out allocation success rate ranged from 45% to 82%; the juveniles of both *L. erythropterus* and *L. malabaricus* achieved higher allocation success rates (82%) compared to adults (45% and 50% for *L. erythropterus* and *L. malabaricus* respectively) (see Supplementary Table S4). The majority of the misclassified samples were allocated to juvenile *L. malabaricus*. The partitioning of diet compositions among the four groups were also evidenced by the observations of each group clustering together on the plot of the first two canonical axes (Fig. 3).

DistLM identified significant effects of species on juvenile diet composition, whereas other sampling variables such as TL, depth, time and date of sampling were not significant as interaction effects (see Supplementary Table S5-A). This finding indicates that the inter-specific dietary partitioning patterns are unlikely to be confounded by other sampling variables. BEST analyses identified that the most parsimonious model describing the diet composition was the model with species as a single variable, which supports the DistLM marginal test results (see Supplementary Table S5-B).

Diet composition of adult *L. malabaricus* was unique from those of other three groups, mainly characterised by the following three factors. Firstly, prey richness was the highest with the total number of prey taxa at 19, whereas those of other groups were between 10 and 13. Secondly, their diet was strongly characterised by a high richness and frequency-of-occurrence of malacostracan taxa (Fig. 2). Thirteen of the 19 prey taxa of adult *L. malabaricus* were malacostracans (accounting for 68%) and all of them except for Decapoda (an order including shrimps and crabs) and *Solenocera* (a genus of prawns) were not shared with the other groups (Fig. 2). Significant influences of malacostracan crustaceans on the partitioning of adult *L. malabaricus* were also indicated by a number of correlation vectors of malacostracan taxa pointing towards the cluster of adult *L. malabaricus* on CAP plot (Fig. 3). These taxa include; *Metapenaeopsis barbata*, *Metapenaeopsis lamellate, Metapenaeopsis* and *Solenocera rathbuni*. frequency-of-occurrence of *S. rathbuni* and *M. barbata* were particularly high, ranging from 42 to 58% (Fig. 2). Thirdly, the patterns of occurrence of teleost taxa were unique from those of the other three groups. For instance, Pomacanthidae was the most common teleost taxa across all the groups but the frequency-of-occurrence were relatively low in adult *L. malabaricus* (67%) compared to those of other three groups (between 91 and 100%) (Fig. 2). In contrast, Carangidae (except *Trachurus*) and *Rastrelliger* were found with 17% frequency-of-occurrence in adult *L. malabaricus* whereas they were not found in other groups (Fig. 2).

Contrary to the diet of adult *L. malabaricus*, which was dominated by malacostracans, no malacostracans was detected in the intestinal content of adult *L. erythropterus* (Fig. 2). Diet composition of adult *L. erythropterus* was characterised by teleosts and soft bodied taxa including tunicates, medusa and comb jellies (Fig. 3). A total of 38% of the prey taxa for adult *L. erythropterus* were teleosts, two of which (Pomacanthidae, *Trachurus barbatus*) had particularly high occurrence rates (100 and 27% respectively; Fig. 2).

Juvenile diet was dominated by teleosts and malacostracans for both *L. erythropterus* and *L. malabaricus*, similar to those of adult *L. malabaricus* (Fig. 2). Eleven and 10 prey taxa were identified from juvenile *L. erythropterus* and *L. malabaricus*, respectively. For juvenile *L. erythropterus*, 36 and 55% of their diet taxa were teleosts and malacostracans respectively, accounting for 91% of their total taxa identified. The following taxa had relatively high frequency-of-occurrence (Fig. 2) and strong correlations with canonical axis 1 pointing towards the cluster of juvenile *L. erythropterus* observations (Fig. 3), indicating the high contribution of the partitioning of their diet: *Euterpina acutifrons, Chlorotocella graciles, Solenocera* and Mysidae. These taxa were either not found or found with a low frequency-of-occurrence in juvenile *L. malabaricus*. For juvenile *L. malabaricus*, 50 and 40% were teleosts and malacostracans respectively. Prey taxa which were unique to *L. malabaricus* juveniles include *Parachaeturichthys polynema*, *Gerres japonicus* and Octopodidae. The frequency-of-occurrence of these taxa were relatively low (9%) (Fig. 2) thus they were not correlated along CAP axes (Fig. 3).

## Discussion

Information on dietary partitioning of sympatric and highly valued fisheries species is fundamental to understanding the ecological processes that support their coexistence. High resolution methods are required to assess dietary partitioning of sympatric, cryptic species, such as *L. erythropterus* and *L. malabaricus*, because they are likely to exhibit subtle differentiation due to their ecological similarity^4,6^. Dietary studies on juvenile fish have also been particularly challenging due to the relatively low abundance and difficulty in identifying small-sized prey items using traditional methods (i.e. morphological identification of gut contents). This study was the first molecular based dietary study on juveniles and adults of sympatric, cryptic fish, which revealed clear dietary partitioning patterns between the species and life history stages.

The major prey categories of adult *L. malabaricus* were teleosts and malacostracan crustaceans which includes crabs, prawns, mantis shrimps and mysid shrimps, whereas no malacostracans were identified in the diet of adult *L. erythropterus*. In contrast, the common prey items of adult *L. erythropterus* were teleosts (specifically pomacanthids, jack mackerel and gobies), in combination with a variety of prey from minor dietary categories, including medusae, tunicates and comb jellies. These findings are supported by those of Salini *et al*.^20^ who also identified a variety of crustaceans from the stomach content of *L. malabaricus*, but not from those of *L. erythropterus*. One of the major factors which constrain and shape diet choice is body morphology such as jaw structure, dentition and body size^53^. This ecomorphology theory may assist in explaining the diet specialization of adult *L. erythropterus* and *L. malabaricus* observed in this study, as these species exhibit notable differences in jaw morphology. *Lutjanus malabaricus* has a longer jaw on average than *L. erythropterus*. One of the common names of *L. malabaricus* and *L. erythropterus* are large-mouth and small-mouth nannygai, respectively, which reflect this diagnostic characteristic. In general, length of fish jaws correlates with the volume of their oral cavity and, consequently, the force of suction^53^ and diversity of prey that they can successfully feed on^54^. The larger mouth of *L. malabaricus* may have increased its feeding capacity, allowing it to prey on a variety of malacostracan crustaceans as observed in the current study and also by previous studies^20^. In contrast, *L. erythropterus*, with a smaller mouth, may have shifted their choice to other prey categories such as medusae, comb jellies and tunicates, which are much slower swimmers and have softer bodies compared to crustaceans. *Lutjanus malabaricus* possesses a larger mean and maximum body length compared to *L. erythropterus*, which is consistent with previous studies^8,20^ and may have played a role as another ecomorphological factor to differentiate the diet patterns.

Habitat associations also play a role in differentiating diet patterns. For example, *L. malabaricus* is considered a true demersal species. In contrast, *in-situ* observations from underwater video indicate that *L. erythropterus* is benthopelagic (S. Newman, C. Wakefield personal observations). This vertical habitat partitioning concurs with the composition of their prey. The major prey categories of adult *L. malabaricus* were malacostracan crustaceans, which are benthic or epibenthic, and prey categories of adult *L. erythropterus* were composed predominantly of teleosts, medusa, tunicates and comb jellies, which generally occupy the water column. These patterns reinforce the idea that diet composition is strongly linked to habitat utilisation, and moreover, diet patterns reflect fine scale partitioning of habitat mosaics between sympatric species.

Another example of the link between habitat association and diet partitioning is the ontogenetic shifts in diet, which was observed in the present studies as well as previous studies on reef fish^9–12^. Juveniles of many coral reef fish, including *L. erythropterus* and *L. malabaricus*, occur in shallow, low-relief habitat such as soft sediments and seagrass beds, whereas adults are found on deeper coral reefs^7,8^. These commonly observed ontogenetic shifts in reef fish diet suggest that life history partitioning is a strategy to provide refuge from predation but also the segregation of resources to minimise intra-specific competition.

While there are previous studies on diet partitioning between life history stages within a species^11–13^ or sympatric fish at the adult stage^4,45^, studies on niche partitioning between juvenile fish of closely related species (i.e. sympatric, cryptic, same genus or family) are extremely rare. Examples include a morphological gut content analysis of *Lutjanus* spp. (i.e. snapper) by Pimentel and Joyeux^55^ and a stable isotope analysis of otoliths of *Albula* spp. (i.e. bonefish) by Haak *et al*.^56^. Although both studies identified significant differences in the measured parameters (diet composition and δ_13_C) between species, diet partitioning of the juvenile stage still remained unclear. Pimentel and Joyeux^55^ reported that their results might have been confounded by other interspecific variations such as length of juvenile fish. Moreover, the stable isotope measured by Haak *et al*.^56^ was not a direct measure of diet composition. Our metabarcoding analyses accounted for other variables that might influence diet, such as the length of the fish, fullness of stomachs, time, date and depth of sampling events. The findings indicted that the diet differentiations within the juvenile stage were solely shaped by the individual species and not by other factors examined, providing the first evidence of diet partitioning between cryptic juveniles of lutjanid species. However, although their diet partitioning was statistically significant, there is still a large proportion of variance unexplained. In addition, this differentiation in diet was not as distinctive as those observed within the adult stage, particularly at the level of general prey category. Contrary to the clear differences in morphology and habitat separation within the adult stage, juveniles of *L. erythropterus* and *L. malabaricus* are visually cryptic and fine scale habitat differentiation has not previously been studied. Further studies are required to better understand niche partitioning mechanisms in juvenile stages of reef fish.

While the interspecific diet compositions of juveniles were analysed with potential confounding factors taken into account, such analyses were not possible for adult samples due to the lack of detailed sampling information. Furthermore, sampling period for juveniles spanned over a month, whereas adult samples were collected on one day thus the adults’ prey compositions identified in this study represented the “meal” of the day rather than the general diet composition of the species. Given that the interspecific diet partitioning patterns of cryptic juveniles were solely influenced by species and not by other factors including sampling dates, it is likely that the diet partitioning between the morphologically distinctive adults were also shaped by the individual species and not confounding factors. However, further studies with an extended sampling period and detailed sampling information are required to resolve this uncertainty.

In the past decade there have been a growing number of studies to assess diet of diverse fauna using DNA metabarcoding approaches due the various advantages associated with this technique. One of the main advantages is the unprecedented level of resolution in prey taxa detection^30,45^. Previous dietary studies on predatory fish using traditional morphological methods achieved high levels of resolution for penaeid (prawn) identification whereas few or no prey teleosts (fish) were identified at species and genus levels^11,57^. In this study, 57% of the prey taxa identified was to species and 19% to genus. For teleosts in particular, 60% of the 10 teleost taxa were identified at species level. Taxonomic levels were dropped from species to genus or even further when multiple species were assigned to one sequence through the LCA assignment algorithm. This is likely to be the result of a lack of comprehensive reference databases, suggesting that improvement to reference databases can further increase the resolution of prey detection via metabarcoding.

Another notable advantage is the high sensitivity of detection of digested prey items. Most traditional dietary studies using morphological identification approaches only investigate prey items in stomach content^10,11,20^ as they are less digested compared to those from intestine and faecal samples. However, stomach contents provide snapshots of prey composition based on items that are ingested immediately before being captured, especially for predators with high a digestion rate. In contrast, metabarcoding techniques are capable of identifying highly digested prey taxa, in some cases, from lower gastrointestinal tracts or even from faecal samples^47,58^. Due to this high level of sensitivity to the detection of highly digested prey, we were able to identify diverse prey taxa from intestinal contents even from individuals with empty stomachs. Furthermore, the fullness of a stomach and sampling time were not significant in the analyses and as such were not influential factors. This study also detected DNA sequences of soft body prey taxa such as comb jellies, tunicates and medusae, possibly due to the increased sensitivity of this method as tissues of soft-bodied taxa can be quickly digested and are difficult to identify visually^59,60^. Such advantages and the associated outcomes identified in this study suggest that metabarcoding is a promising tool for dietary studies, particularly to analyse partitioning between closely associated species where fine-scale resolution is required.

Along with the advantages provided by DNA metabarcoding, there are also limitations associated with this technique. A common challenge encountered in molecular based dietary analyses is to extract and amplify prey-DNA since host-DNA is frequently amplified and often masks highly degraded prey-DNA^61,62^. We were able to overcome these challenges and successfully sequenced prey-DNA by applying host-specific blocking primers which were designed and tested during this study. Another metabarcoding shortcoming is the limited applications for quantitative analyses. For instance, the weight of prey items are often recorded in traditional dietary studies^10,20,22^, however, the utility of relative read abundance (RRA) from metabarcoding data as a proxy for relative abundance of prey taxa is still under debate^44–46,58^. RRA was explored in this study but ultimately deemed to have too many potential flaws – notably, RRA can be particularly biased when blocking primers or universal primers are applied^48^, which was the case in our study. Size of the prey item is another piece of information that cannot be confirmed using a metabarcoding approach. Dietary studies using traditional morphological approaches also often reveal how the size of prey items can increase, as the size of the consumer grows from a juvenile to an adult^11,12^. This is not feasible with metabarcoding data. The prey taxa detected in this study could be from any life history stage including larvae and eggs. Lack of comprehensive DNA databases can lead to the misidentification of prey taxa and/or reduced taxonomic resolution via LCA assignment algorithm. Other sources of metabarcoding limitations include PCR bias^63^, missing prey identification due to a lack of its sequence in reference databases^64^, and the detection of secondary prey (the prey of a prey)^65^.

In conclusion, high resolution dietary studies of sympatric species provide a tool to better understand not only trophic links, but also ecological and evolutionary processes, behaviour, essential habitat associations, and ultimately management strategies. Our dietary studies with metabarcoding approach identified significant differences in diet composition of juvenile and adult *L. erythropterus* and *L. malabaricus*. Firstly, given that our study species are phylogenetically, morphologically and ecologically closely-allied forms, our findings provide us with new insights on the specialled adaptations required for the coexistence of closely related species. In particular, this study provides the first evidence of diet partitioning between cryptic juveniles of different snapper species. Secondly, this study provides an increased understanding of the dietary preferences of both adults and juveniles of important fishery species. Combined with the close association between diet and habitat, the different diet compositions may imply differential vulnerability to a changing environment. For instance, *L. malabaricus* adults, whose primary diets consisted of benthic malacostracan crustaceans, may be more susceptible to habitat degradation than *L. erythropterus* adults, who predominantly feed on diet items presumably suspended in the water column. Finally, our study highlights the robustness of DNA metabarcoding to detect fine-scale differences in diet between sympatric species, including the detection of soft bodied prey. While this study examined two sympatric species in both juvenile and adult forms, it also facilitates a wider ‘whole of ecosystem’ view of how these species (and their prey) coexist within the wider trophic network. Ultimately, these data may enable more informed fisheries management decisions in regard to spatial management arrangements and also how these species may respond to future climatic perturbations. Furthermore, the selection of a range of fish taxa that profile a wide diversity of the ecosystem (via their diet) may form the basis of a monitoring program that extends beyond just fish into whole of ecosystem responses.

## Supplementary information

Supplementary information.

## Data Availability

The datasets analysed during the current study are available from the corresponding author on reasonable request.
