# Supplementary material for: Partitioning of diet between species and life history stages of sympatric and cryptic snappers (Lutjanidae) based on DNA metabarcoding
Source: Sci Rep. 2020 Mar 9;10:4319. doi: 10.1038/s41598-020-60779-9 (PMC7062689; doi:10.1038/s41598-020-60779-9)
Supplement: Supplementary file 1 — Supplementary information. [file 41598_2020_60779_MOESM1_ESM.pdf]

Supplementary information for :
Partitioning of diet between species and life history stages of sympatric and cryptic snappers (Lutjanidae) based on DNA metabarcoding

Miwa Takahashi[1]*, Joseph D. DiBattista[1,2], Simon Jarman[1, 3], Stephen J. Newman[4], Corey B. Wakefield[4], Euan S. Harvey[1], Michael Bunce[1, 5]

1 School of Molecular and Life Sciences, Curtin University, Kent Street, Bentley, Perth, WA, 6102, Australia

2 Australian Museum Research Institute, Australian Museum, 1 William Street, Sydney, NSW, 2010, Australia

3 School of Biological Sciences, University of Western Australia, 35 Stirling Highway, Perth, WA, 6009, Australia

4 Western Australian Fisheries and Marine Research Laboratories, Department of Primary Industries and Regional Development, Government of Western Australia, P.O. Box 20, North Beach, WA, 6920, Australia

5 Environmental Protection Authority, 215 Lambton Quay, Wellington 6011, New Zealand

* Corresponding author: miwatakahashi582@gmail.com

This file contains the following supplementary materials.

"Blocking primer development"

Table S1. Fish16S assay and the host-specific blocking primer sequences (LEBP, *Lutjanus erythropterus* blocking primer; LMBP, *L. malabaricus* blocking primer).

Figure S1. Frequency histogram of the number of base pairs mismatched with *Lutjanus erythropterus* blocking primer (LEBP) (left) and *L. malabaricus* blocking primer (LMBP) (right), based on in-silico examination of 16S ribosomal RNA (rRNA) sequences.

Figure S2. Mean C_T values (+/- SD, standard deviation) at different amount of *Lutjanus malabaricus* blocking primer (LMBP) and annealing temperatures.

Figure S3. Relative read abundance of prey (*Sardinops sagax*) and host (*Lutjanus erythropterus* and *L. malabaricus*) DNA with/without blocking primers.

Figure S4. The quality profiles of forward (A) and reverse (B) reads of paired-end sequences.

"Assessment of sampling and sequencing depth"

Figure S5. Mean number of reads (A) and mean number of prey taxa (B) (+/- SD, standard deviation) obtained from the intestinal content of adult and juvenile *Lutjanus erythropterus* and *L. malabaricus*.

Figure S6. Rarefaction curves of intestinal content samples from a universal (18SUni) and three sets of taxa specific assays (Fish16S, SCrust, and SCeph).

Figure S7. Species accumulation curves of amplicon sequence variants (ASVs) (A) and prey taxa (B) identified from the intestinal contents of adult and juvenile *Lutjanus erythropterus* (LE) and *L. malabaricus* (LM) with all assays combined.

Table S2. Taxonomic assignment resolutions (A) and the results of PERMANOVA (B), pairwise-PERMANOVA (C) using different percent identify match thresholds (0, 1 and 2%).

Table S3. The list of prey taxa as lowest common ancestors (LCA) and number of ASV, species, genus and family which were assigned to the LCA.

Table S4. The results of cross validation of diet compositions observations in the canonical analyses (CAP) ordination, following to the leave-one-out approach.

Table S5. The results of distance based linear model (DistLM) to test the effects of sample variability on juvenile diet composition.

References

Blocking primer development

A difficulty in DNA-based diet studies is when PCR favours the amplification of the higher quality host (i.e. predator) DNA over partially digested prey DNA, and consequently the rare sequences of prey DNA may not be represented [1–4]. Indeed, our pilot study identified only host species' sequences using Fish16S assay even when obvious prey fish tissue (from other species) was found in their stomachs. Therefore, we designed species-specific blocking primers (LEBP and LMBP for *L. erythropterus* and *L. malabaricus*, respectively) to suppress the amplification of host DNA. LEBP and LMBP consisted of: 1) the 10 bases of the 3' end of Fish16S forward primer; 2) the 15 bases that were specific to each of the host sequences; and 3) a C3 spacer CPG at the 3' end (Table S1). The 15 bases of host-specific sequences bind to the host sequences, and the C3 spacer inhibit its amplification. We conducted *in-silico* and *in-vitro* experiments to test the efficacy of the blocking primers and identify the optimal PCR protocols.

In-silico examination of 16S ribosomal RNA (rRNA) sequences was conducted in order to validate that the blocking primers would only inhibit the amplification of host-DNA and not prey-DNA. In other words, the 15 base pairs of the designed blocking primers needed to be host-specific and different from prey sequences. A total of 65 species of fish (at the order- or family-level) were listed as potential prey in the diet of the two snapper species based on Salini et al. [5]; 80 reference sequences of the 65 species were available from an in-house fish database [6]. *Sardinops sagax* was also included in the potential prey list even though its sequence were not available from this in-house database because it is commonly used as a bait in the trap fisheries in Western Australia [7]. For *S. sagax*, all the 40 available reference sequences from the NCBI reference database (downloaded 10 July 2017) were tested against the blocking primers. The results of the in-silico study showed that the number of base pairs mismatched with the blocking primers ranged between 1 and 8 (Fig. S1). On average 3.3 ± 1.29 (SD) base pairs and 3.99 ± 1.23 base pairs mismatched with host-specific regions of LEBP and LMBP, respectively. None of the prey sequences matched to the blocking primers with 100% fidelity. This mismatch with prey and fidelity with hosts suggests that the blocking primers should block the host-DNA and would not interfere the amplification of target prey-DNA. However, Piñol et al. [8] suggested that blocking primers could co-block non-target species with as little as four mismatches during PCR reactions, suggesting the limitation of a quantitative analysis. Given these limitations in quantitative approaches, we carried out multivariate analyses using PA data only. The software Geneious 10.2.3 was used for all alignments [9].

The first in-vitro pilot study was conducted to test the optimal annealing temperatures and concentration of the blocking primers. Firstly, DNA was extracted from the fin clips of two *L. malabaricus* using DNeasy Blood & Tissue Kits following the manufacturer's protocol (QIAGEN, CA, USA) to provide a high quality template for downstream amplification. Quantitative PCR (qPCR) was carried out on these DNA extracts, using the three PCR mixtures containing different amount of LMBP. Three different annealing temperatures (50, 54, and 58°C) were also tested for each of the PCR mixtures. All PCR mixtures contained 2 µL of DNA extract and 23 µL of PCR mastermix, which consisted of 2 µL of 25mM $MgCl_2$ solution (Applied Biosystems, CA, USA), 2.5 µL of 10x Taq Gold buffer (Applied Biosystems), 1 µL of 10mg/ml Bovine Serum Albumin (Fisher Biotec, WA, Australia), 1 µL each of forward and reverse Fish16S assay (10µM), 0.25 µL of 25mM dNTPs (Astral Scientific, NSW, Australia), 0.6 µL of 1/10,000 SYBR Green dye (Life Technologies, CA, USA), and 0.2 µL of Taq polymerase Gold (Applied

Biosystems). Different amounts of LMBP were added to each of the PCR mixtures to test their efficacy; 1 µL of 100 µM LMBP (10 times higher than Fish16S assay), 1 µL of 200 µM LMBP (20 times higher than Fish16S assay), and no blocking primer. Ultrapure water was added to the PCR mixture to bring the reaction volume up to 23 µL per sample. qPCRs were performed in 25 µL reaction volumes containing 2 µL of DNA extract and 23 µL of PCR mastermix using a StepOnePlus Real-Time PCR System (Applied Biosystems), using the following cycling program: (i) 95°C for 5 minutes, (ii) 45 amplification cycles of 95°C for 30 seconds, (iii) three different annealing temperatures (50, 54, and 58 °C) for 30 seconds, and (iv) 72°C for 45 seconds, and (v) a final extension step at 72°C for 10 minutes. Cycle threshold (Ct) values were recorded from the amplification curves and compared to assess the efficiency of the blocking. primer. Two-way ANOVA was used to test the significance of the two factors (the amounts of LMBP added and annealing temperature) on the average C_T values using the software RStudio (v.1.0.143, https://rstudio.com/) [10]. The mean C_T values of *L. malabaricus* DNA template qPCR were significantly higher when LMBP was applied in the PCR mixture (two-way ANOVA; $F_{(2,17)} = 173.55$, $p < 0.0001$) whereas there was no difference between the LMBP to Fish16S ratio of 10 to 1 and 20 to 1 (Fig. S2). This indicates that LMBP effectively reduced the amplification of *L. malabaricus* DNA but adding extra LMBP did not improve the effectiveness. The annealing temperature had no significant effect on C_T values (two-way ANOVA ; $F_{(2,17)} = 4.39$, $p = 0.05$), and there was no significant interaction between the two factors (two-way ANOVA ; $F_{(4,17)} = 1.69$, $p = 0.24$) (Fig. S2). Based on these results, downstream PCRs were carried out with the PCR reaction containing 10 times more blocking primer than Fish16S assay (1 µL of 100 µM blocking primer to 1 µL of 10 µM Fish16S assay in each PCR reaction), with an annealing temperature of 58°C.

The second in-vitro pilot study was carried out on the mock samples containing host- and prey-DNA mixed at different ratios. Firstly, qPCR was carried out with the DNA extracted from the fin clips of *L. erythropterus*, *L. malabaricus*, and *S. sagax* using Fish16S assay without blocking primers, and the relative concentration of their DNA templates were estimated using their C_T values. DNA extracts were added to create the mock samples with different ratios of prey- to host-DNA (1:1, 1:100, 1:1000, and 10:1). The mock samples were amplified with and without blocking primers and sequenced each with unique six to eight base pair multiplex identifier (MID) tags in duplicate. Unique combinations of forward and reverse MID tags allowed us to assign sequences to a sample after metabarcoding the pooled samples. Single-end sequencing was performed for the pooled amplicons using an Illumina MiSeq platform in the Trace and Environmental DNA (TrEnD) Laboratory at Curtin University in Western Australia, following manufacture's protocols. Version 2 reagent kit and either Standard or Nano flow cell were used for 300 – 500 cycles. Both LEBP and LMBP effectively inhibited the amplification of host-DNA and increased the chance of detecting prey DNA when the prey-host DNA ratios were 1 to 1, 1 to 100, and 10 to 1 (Fig. S3). When the *L. erythropterus* DNA was 1000 times higher than the *S. sagax* DNA, LEBP was no longer able to suppress the amplification of host-DNA in order to detect the prey DNA (Fig. S3).

Following these results, downstream PCRs were carried out with the PCR reaction containing 10 times more blocking primer than Fish16S assay (1 µL of 100 µM blocking primer and 1 µL of 10 µM Fish16S assay in each PCR reaction), with the annealing temperature of 58°C.

Table S1. Fish16S assay and the host-specific blocking primer sequences (LEBP, *Lutjanus erythropterus* blocking primer; LMBP, *L. malabaricus* blocking primer). Fish16S target teleost sequences at 16S rRNA mitochondrial gene region. The first 10 base pairs of the blocking primers (bold and italic font) overlap with the 3' end of the forward Fish16S assay, followed by 15 base pairs of the host specific sequences. The C3 spacer at the 3' end is a modified DNA oligonucleotide, which inhibits annealing.

Primer	Primer sequence (5' – 3')
**Fish16S (forward)**	GACCCTATGG***AGCTTTAGAC***
**Fish16S (reverse)**	CGCTGTTATCCCTADRGTAACT
**LEBP**	***AGCTTTAGAC***ACCAAGGCAGA**C**CAT / **C3** /
**LMBP**	***AGCTTTAGAC***ACCAAGGCAGA**A**CAT / **C3** /

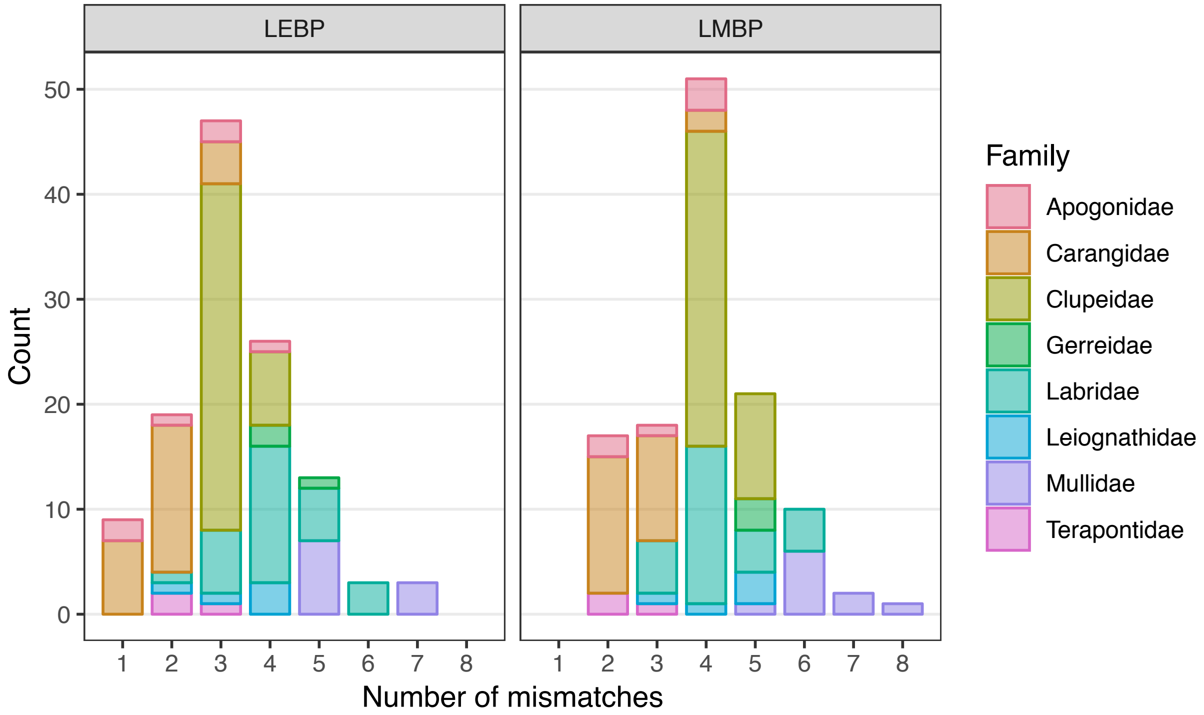

Figure S1. Frequency histogram of the number of base pairs mismatched with *Lutjanus erythropterus* blocking primer (LEBP) (left) and *L. malabaricus* blocking primer (LMBP) (right), based on in-silico examination of 16S ribosomal RNA (rRNA) sequences.

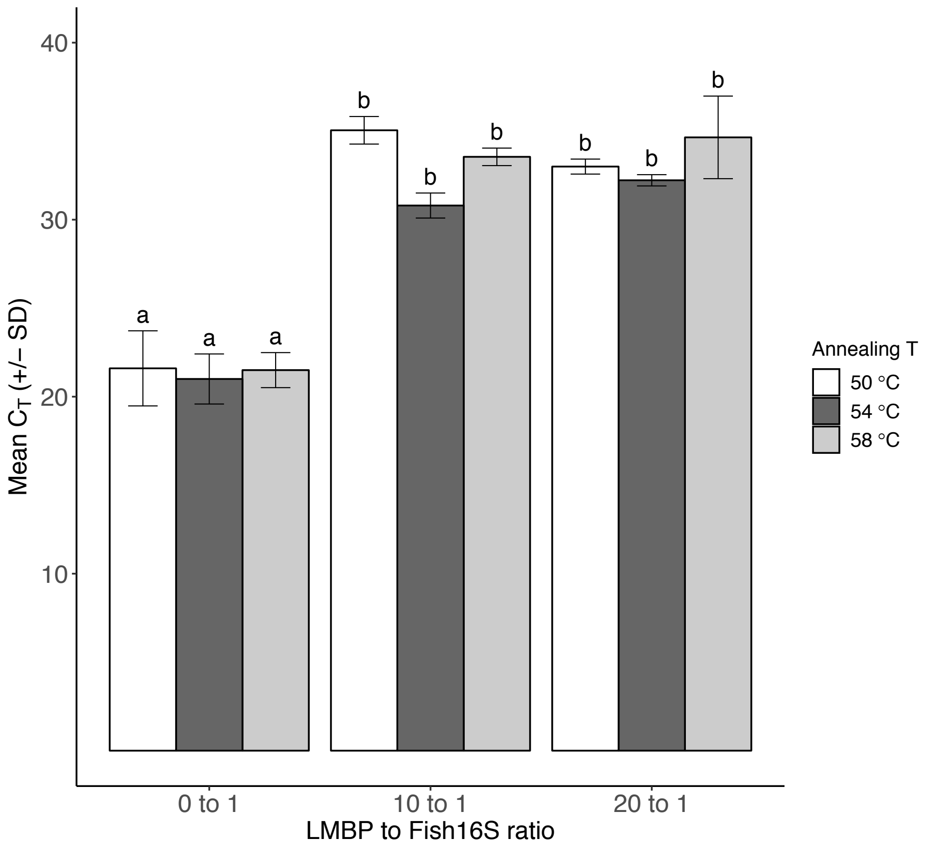

Figure S2. Mean C_T values (+/- SD, standard deviation) at different amount of *Lutjanus malabaricus* blocking primer (LMBP) and annealing temperatures. The ratio on the x-axis refers to the ratio of LMBP to Fish16S assay added into the PCR mastermix. Letters above each bar imply statistically similar means for C_T values.

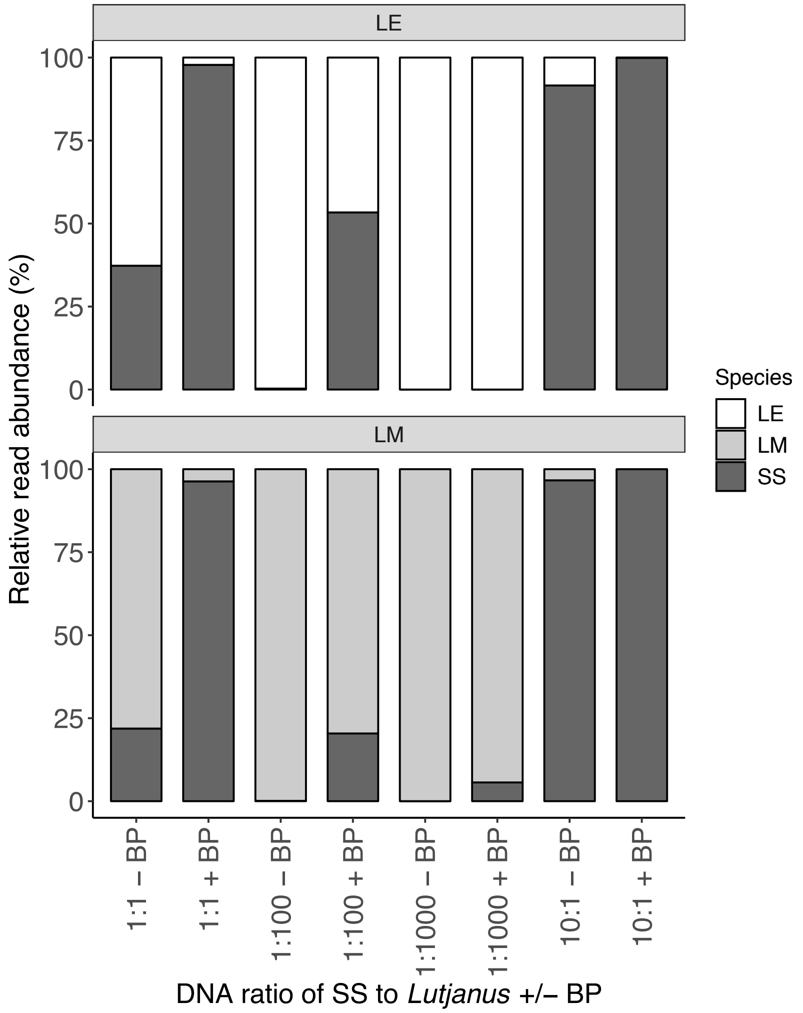

Figure S3. Relative read abundance of prey (*Sardinops sagax*) and host (*Lutjanus erythropterus* and *L. malabaricus*) DNA with/without blocking primers. The ratio on the x-axis refers to the DNA ratio of prey to host in the template. Negative and positive symbols indicate mastermixes without and with blocking primer, respectively. SS, *S. sagax*; LE, *L. erythropterus*; LM, *L. malabaricus*; BP, blocking primer.

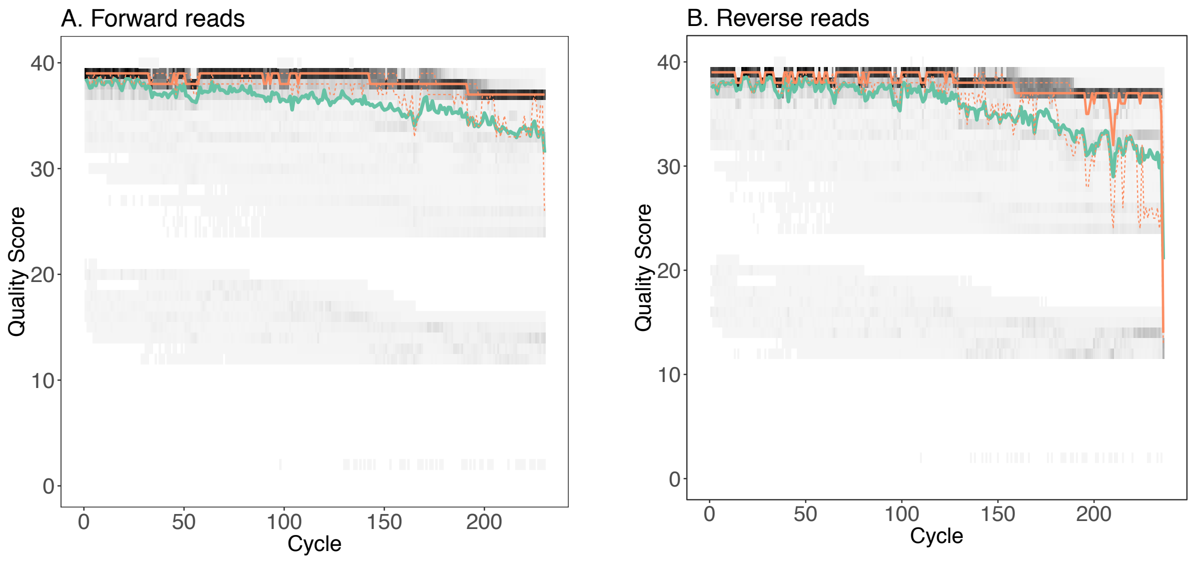

Figure S4. The quality profiles of forward (A) and reverse (B) reads of paired-end sequences. Green line, orange line, and dashed orange lines represent the mean, median, and the 25th and 75th quantiles respectively. A grey-scale heat map represents the distribution of quality scores at each position, with dark colours corresponding to higher frequency. The mean quality scores gradually declined throughout the cycles for forward reads, whereas reverse reads experienced a steeper decline in quality scores approximately after the 100th cycle.

Assessment of sampling and sequencing depth

The relationship between number of reads and prey taxa was examined using Pearson correlation in order to inspect whether the number of prey taxa detected was affected by sequencing depth. Analyses of variance (ANOVA) was also carried out to test the differences in mean number of reads and prey taxa detected between each group. The assumption of the samples coming from a normal distribution was tested and the number of prey taxa were transformed using a square-root transformation to meet the assumption. Rarefaction curves were plotted describing the diet of each species and life history stage. Random subsampling of sequences was conducted 1000 times at every 1000 reads for each sample, following to the approach explained by Colwell [11], and the total number of ASV and prey taxa detected by subsampling were averaged within the samples of the same group (species and life history stage). Species accumulation curves were plotted to assess whether the number of samples were sufficient to capture the majority of their potential prey taxa consumed by each species and life history stage. The software RStudio (v.1.0.143, https://rstudio.com/) was used to carry out statistical analyses and subsampling, and produce plots [10].

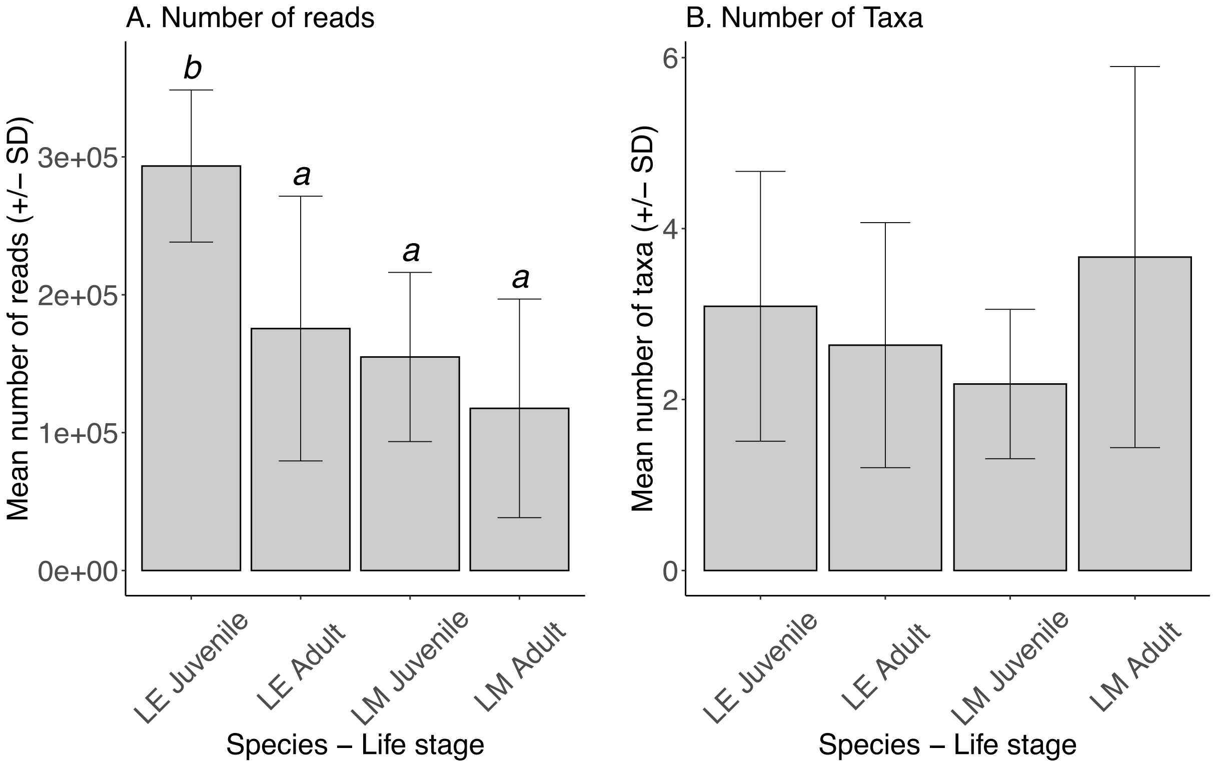

Figure S5. Mean number of reads (A) and mean number of prey taxa (B) (+/- SD, standard deviation) obtained from the intestinal content of adult and juvenile *Lutjanus erythropterus* and *L. malabaricus*. Italics letters above the error bars imply statistically similar means for number of reads.

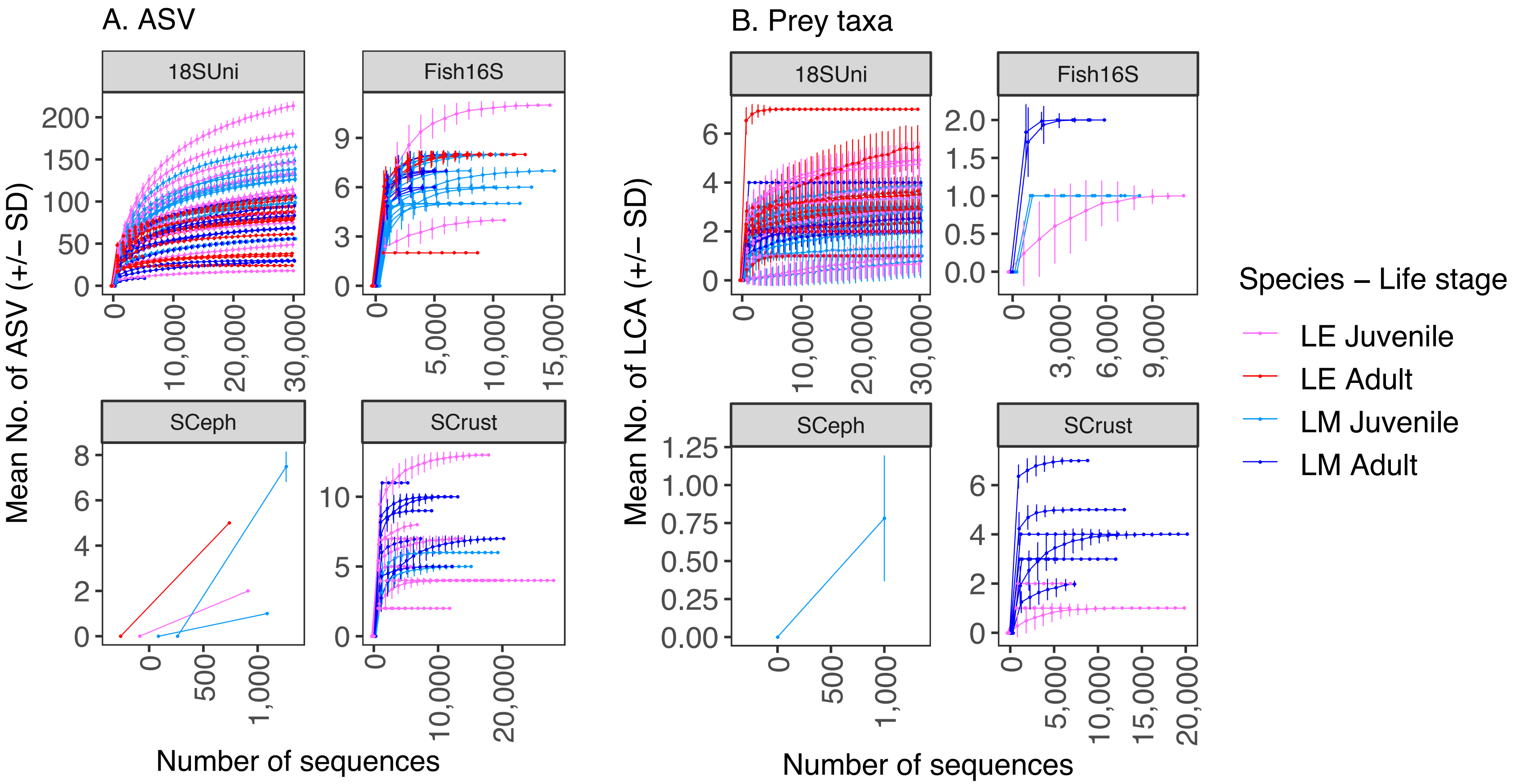

Figure S6. Rarefaction curves of intestinal content samples from a universal (18SUni) and three sets of taxa specific assays (Fish16S, SCrust, and SCeph). The plateaus of the curves indicate the sufficient sequencing efforts to reveal the majority of the detected amplicon sequence variants (ASVs) (A) and prey taxa (B). SD, standard deviation.

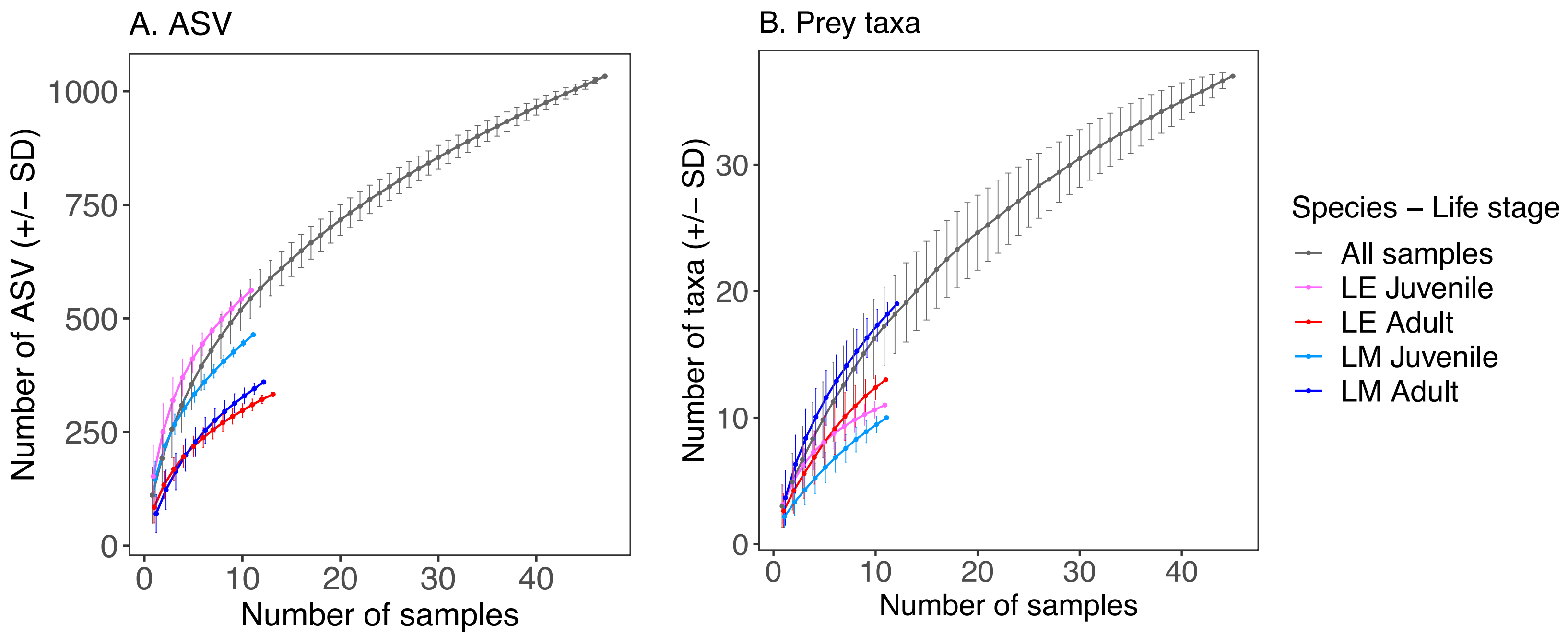

Figure S7. Species accumulation curves of amplicon sequence variants (ASVs) (A) and prey taxa (B) identified from the intestinal contents of adult and juvenile *Lutjanus erythropterus* (LE) and *L. malabaricus* (LM) with all assays combined. SD, standard deviation.

Table S2. Taxonomic assignment (A) as well as the results of PERMANOVA (B) and pairwise-PERMANOVA (C) using different percent identify match thresholds (0, 1 and 2%). The threshold defines the maximum difference between the percent identity matches of primary and non-primary reference sequences allowed in the lowest common ancestor (LCA) assignment algorithm. When the difference between the percent identity matches of primary and non-primary reference sequences was more than the threshold, the non-primary reference sequences were omitted prior to LCA assignment. PERMANOVA (B) and pairwise-PERMANOVA (C) examined the differences in diet composition of juvenile and adult *Lutjanus erythropterus* (LE) and *L. malabaricus* (LM), using 9999 permutations. Fullness of stomach was incorporated into the analyses as a covariate in order to test its effect on diet composition. The tests were based on Jaccard coefficient matric for presence and absence (PA) datasets.

A. Taxonomic assignment

	Number of prey taxa				
Threshold (%)	Total	Species level	Genus level	Family level	Order level
0	51	35 (69%)	10 (19%)	4 (8%)	2 (4%)
1	37	21 (57%)	7 (19%)	6 (16%)	3 (8%)
2	37	20 (54%)	8 (22%)	6 (16%)	3 (8%)

B. PERMANOVA

	Threshold (%)	df	SS	F Model	p
Species	0	1	8943	3.14	0.0002
	1	1	6416	2.40	0.0039
	2	1	6314	2.36	0.0044
Life stage	0	1	7715	2.71	0.0012
	1	1	7667	2.87	0.0008
	2	1	7880	2.95	0.0008
Fullness of stomach	0	1	4566	1.60	0.0644
	1	1	3820	1.43	0.1161
	2	1	3803	1.42	0.1222
Species x Life stage	0	1	7954	2.79	0.0022
	1	1	8071	3.02	0.0025
	2	1	8213	3.08	0.0013
Residuals	0	40	113960		
	1	40	106770		
	2	40	106800		

C. Pairwise-PERMANOVA

		Threshold (%)	df	t	p
Inter-specific	Within Juvenile	0	19	1.59	0.0049
		1	19	1.50	0.0189
		2	19	1.50	0.0201
	Within Adult	0	20	1.61	0.0034
		1	20	1.43	0.0269
		2	20	1.40	0.0283
Intra-specific	Within LE	0	19	1.51	0.0199
		1	19	1.43	0.0313
		2	19	1.43	0.0305
	Within LM	0	20	1.83	0.0023
		1	20	1.99	0.0019
		2	20	2.02	0.0014

Table S3. The list of prey taxa as lowest common ancestors (LCA) and number of ASV, species, genus and family which were assigned to the LCA. % match indicate the range of % similarity between each ASV and the reference sequences of assigned taxa. Where LCA taxa level is genus or higher, the species list contains more than one species with a common ancestor.

LCA	LCA level	Primer	No. ASV	No. species	No. genus	No. family	% match	Species list
Rastrelliger	genus	Fish16S	1	2	1	1	99.52 ~ 100	*Rastrelliger kanagurta/ R. brachysoma*
Auxis rochei	species	18SUni	4	1	1	1	98.25 ~ 100	*Auxis rochei*
Pomacanthidae	family	18SUni	104	10	4	1	95.04 ~ 98.25	*Apolemichthys griffisi/ Centropyge flavissima/ Holacanthus tricolor/ Centropyge eibli/ C. venusta/ C. aurantia/ Paracentropyge multifasciata/ Holacanthus ciliaris/ H. passer/ H. africanus*
Labrus bergylta	species	18SUni	4	1	1	1	95.75 ~ 98	*Labrus bergylta*
Tridentiger barbatus	species	18SUni	1	1	1	1	95.01	*Tridentiger barbatus*
Parachaeturichthys polynema	species	Fish16S	1	1	1	1	97.49	*Parachaeturichthys polynema*
Carangidae	family	Fish16S	1	2	2	1	100	*Decapterus maruadsi/ Trachurus novaezelandiae*
Trachurus	genus	18SUni	1	2	1	1	95.24	*Trachurus mediterraneus/ T. trachurus*
Gerres filamentosus	species	Fish16S	1	1	1	1	98.04	*Gerres filamentosus*
Gerres japonicus	species	Fish16S	1	1	1	1	97.17	*Gerres japonicus*
Salpidae	family	18SUni	2	5	3	1	95.29 ~ 96.34	*Ritteriella retracta/ Iasis cylindrica/ Salpa fusiformis/ S. thompsoni/ S. maxima*
Iasis cylindrica	species	18SUni	1	1	1	1	100	*Iasis cylindrica*
Cyclosalpa	genus	18SUni	1	3	1	1	99.22 ~ 100	*Cyclosalpa sewelli/ C. polae/ C. affinis*
Megalocercus huxleyi	species	18SUni	1	1	1	1	99.47	*Megalocercus huxleyi*
Oratosquilla oratoria	species	SCrust	1	1	1	1	95.43	*Oratosquilla oratoria*

Mysidae	family	18SUni	1	5	4	1	95.65 ~ 96.42	*Boreomysis arctica/ B. tridens/ Mysidella typica/ Dactylerythrops bidigitata/ Rhopalophthalmus sp.*
Decapoda	order	18SUni	14	30	25	14	96.68 ~ 100	*Daldorfia horrida/ Pachygrapsus fakaravensis/ Eriphia smithii/ E. sebana/ E. verrucosa/ Portunus trituberculatus/ Otmaroxanthus balboai/ Pseudocarcinus gigas/ Panopeus herbstii/ Platyxanthus orbignyi/ Pilumnus floridanus/ Menippe nodifrons/ M. mercenaria/ M. adina/ Lobopilumnus agassizii/ Callinectes sapidus/ Carpilius maculatus/ Frevillea barbata/ Maja crispata/ M. brachydactyla/ Zaops ostreum/ Tritodynamia horvathi/ Eriocheir sinensis/ Homalaspis plana/ Hypothalassia armata/ Hexapanopeus angustifrons/ Eupilumnus laciniatus/ Eriphides hispida/ Charybdis japonica/ Ozius rugulosus*
Solenocera	genus	SCrust	1	2	1	1	95.35	*Solenocera crassicornis/ S. pectinata*
Solenocera rathbuni	species	SCrust	1	1	1	1	98.26	*Solenocera rathbuni*
Processa japonica	species	SCrust	1	1	1	1	95.91	*Processa japonica*
Monomia argentata	species	SCrust	1	1	1	1	100	*Monomia argentata*
Lupocyclus philippinensis	species	SCrust	1	1	1	1	98.28	*Lupocyclus philippinensis*
Trachysalambria nansei	species	SCrust	1	1	1	1	100	*Trachysalambria nansei*
Trachypenaeus curvirostris	species	SCrust	1	1	1	1	95.35	*Trachypenaeus curvirostris*
Metapenaeopsis	genus	SCrust	1	2	1	1	95.93	*Metapenaeopsis barbata/ M. acclivis*
Metapenaeopsis palmensis	species	SCrust	1	1	1	1	98.26	*Metapenaeopsis palmensis*

Metapenaeopsis lamellata	species	SCrust	1	1	1	1	97.67	*Metapenaeopsis lamellata*
Metapenaeopsis barbata	species	SCrust	5	1	1	1	95.32 ~ 98.83	*Metapenaeopsis barbata*
Chlorotocella gracilis	species	SCrust	1	1	1	1	98.13	*Chlorotocella gracilis*
Ogyrides	genus	18SUni	1	1	1	1	96.72	*Ogyrides sp.*
Alpheidae	family	18SUni	5	4	2	1	97.47 ~ 98.23	*Alpheus packardii/ A. lobidens/ Alpheidae sp.*
Euterpina acutifrons	species	18SUni	2	1	1	1	99.74 ~ 100	*Euterpina acutifrons*
Candacia truncata	species	18SUni	8	1	1	1	99.47 ~ 100	*Candacia truncata*
Octopodidae	family	SCeph	1	3	2	1	95.83	*Cistopus taiwanicus/ Octopus fusiformis/ Octopus sp*
Eunicida	order	18SUni	4	2	2	2	99.22 ~ 99.74	*Chloeia flava/ Archinome rosacea*
Leptothecata	order	18SUni	1	4	3	3	98.20 ~ 98.72	*Octophialucium indicum/ Blackfordia virginica/ Aequorea victoria/ A. aequorea*
Lyrocteis	genus	18SUni	1	1	1	1	100	*Lyrocteis sp.*

Table S4. The results of cross validation of diet compositions observations in the canonical analyses (CAP) ordination, following to the leave-one-out approach. The values are the number of samples allocated into each of the classified group. The juveniles of both *Lutjanus erythropterus* (LE) and *L. malabaricus* (LM) achieved higher allocation success rates (82%) versus adults. The allocation success rates of adults LE and LM were 45 % and 50 %, respectively, and the majority of the misclassified samples were allocated to LM Juvenile.

		Classified group					
		LE Juvenile	LE Adult	LM Juvenile	LM Adult	Total	% correct
Original group	LE Juvenile	9	0	2	0	11	82
	LE Adult	0	5	5	1	11	45
	LM Juvenile	2	0	9	0	11	82
	LM Adult	0	1	5	6	12	50

Table S5. The results of distance based linear model (DistLM) to test the effects of sample variability on juvenile diet composition. Marginal test results (A) shows the significance levels of each variable on diet compositions. BEST solution results (B) summarised the top five, most parsimonious combination of variables that best explained the juvenile diet composition based on the Akaike Information Criterion with finite sample sizes (AICc). The tests were based on a Jaccard coefficient matrices for presence and absence (PA) datasets. TL = total length (mm).

A. Marginal tests

**Variables**	**df**	**F**	**p**
Species	2	2.11	0.028
TL	2	0.46	0.91
Depth	2	0.17	0.99
Time	6	1.08	0.25
Date	3	0.81	0.61

B. BEST solutions

**AICc**	**R^2**	**Selected variables**
174.07	0.096	Species
175.41	0.039	date
175.78	0.023	TL
175.81	0.13	Species, date
176.1	0.008	depth

References

1. Su, M., Liu, H., Liang, X., Gui, L. & Zhang, J. Dietary analysis of marine fish species: Enhancing the detection of prey-specific DNA sequences via high-throughput sequencing using blocking primers. *Estuaries and Coasts* **41**, 560–571 (2017).
2. Vestheim, H. & Jarman, S. N. Blocking primers to enhance PCR amplification of rare sequences in mixed samples - A case study on prey DNA in Antarctic krill stomachs. *Front. Zool.* **5**, 1–11 (2008).
3. Leray, M., Agudelo, N., Mills, S. C. & Meyer, C. P. Effectiveness of annealing blocking primers versus restriction enzymes for characterization of generalist diets: Unexpected prey revealed in the gut contents of two coral reef fish species. *PLoS One* **8**, e58076 (2013).
4. Vestheim, H., Deagle, B. E. & Jarman, S. N. Application of blocking oligonucleotides to improve signal-to-noise ratio in a PCR. in *PCR Protocols. Method in Molocular Biology (Methods and Protocols)* (ed. Park, D. J.) **687**, 265–274 (Humana Press, 2011).
5. Salini, J. P., Blaber, S. J. M. & Brewer, D. T. Diets of trawled tredatory fish of the Gulf of Carpentaria, Australia, with particular reference to predation on prawns. *Aust. J. Mar. Freshw. Res.* **45**, 397–411 (1994).
6. Nester, G. *et al.* Development and evaluation of fish eDNA metabarcoding assays facilitates the detection of cryptic seahorse taxa (family: Syngnathidae). Manuscript submitted for publication. (2019).
7. Newman, S. J. *et al.* Assessment of the potential impacts of trap usage and ghost fishing on the Northern Demersal Scalefish Fishery. *Rev. Fish. Sci.* **19**, 74–84 (2011).
8. Piñol, J., Mir, G., Gomez-Polo, P. & Agustí, N. Universal and blocking primer mismatches limit the use of high-throughput DNA sequencing for the quantitative metabarcoding of arthropods. *Mol. Ecol. Resour.* **15**, 819–830 (2015).
9. Drummond, A. J. *et al.* Geneious v5.1. (2010).
10. RStudio Team. RStudio: Integrated Development for R. (2016).
11. Colwell, R. K. EstimateS: Statistical estimation of species richness and shared species from samples. Version 9. User's Guide. (2013).
